# Nup133 and ERα mediate the differential effects of hyperoxia-induced damage in male and female OPCs

**DOI:** 10.1186/s40348-020-00102-8

**Published:** 2020-08-25

**Authors:** Donna Elizabeth Sunny, Elke Hammer, Sebastian Strempel, Christy Joseph, Himanshu Manchanda, Till Ittermann, Stephanie Hübner, Frank Ulrich Weiss, Uwe Völker, Matthias Heckmann

**Affiliations:** 1 Department of Neonatology and Pediatric Intensive Care, University of Medicine Greifswald, Ferdinand-Sauerbruchstrasse, 17475 Greifswald, Germany; 2 Department of Functional Genomics, University of Medicine Greifswald, Greifswald, Germany; 3 Microsynth AG, Balgach, Switzerland; 4 Department of Pharmacology, Center of Drug Absorption and Transport (C_DAT), University of Medicine Greifswald, Greifswald, Germany; 5 Department of Bioinformatics, University of Medicine Greifswald, Greifswald, Germany; 6 Institute for Community Medicine, University of Medicine Greifswald, Greifswald, Germany; 7 Department of Internal Medicine A, University of Medicine Greifswald, Greifswald, Germany

**Keywords:** White matter damage, Hyperoxia, Preterm brain, Steroid hormones, Sex difference

## Abstract

**Background:**

Hyperoxia is a well-known cause of cerebral white matter injury in preterm infants with male sex being an independent and critical risk factor for poor neurodevelopmental outcome. Sex is therefore being widely considered as one of the major decisive factors for prognosis and treatment of these infants. But unfortunately, we still lack a clear view of the molecular mechanisms that lead to such a profound difference. Hence, using mouse-derived primary oligodendrocyte progenitor cells (OPCs), we investigated the molecular factors and underlying mechanisms behind the differential response of male and female cells towards oxidative stress.

**Results:**

We demonstrate that oxidative stress severely affects cellular functions related to energy metabolism, stress response, and maturation in the male-derived OPCs, whereas the female cells remain largely unaffected. CNPase protein level was found to decline following hyperoxia in male but not in female cells. This impairment of maturation was accompanied by the downregulation of nucleoporin and nuclear lamina proteins in the male cells. We identify Nup133 as a novel target protein affected by hyperoxia, whose inverse regulation may mediate this differential response in the male and female cells. Nup133 protein level declined following hyperoxia in male but not in female cells. We show that nuclear respiratory factor 1 (Nrf1) is a direct downstream target of Nup133 and that Nrf1 mRNA declines following hyperoxia in male but not in female cells. The female cells may be rendered resistant due to synergistic protection via the estrogen receptor alpha (ERα) which was upregulated following hyperoxia in female but not in male cells. Both Nup133 and ERα regulate mitochondrial function and oxidative stress response by transcriptional regulation of Nrf1.

**Conclusions:**

These findings from a basic cell culture model establish prominent sex-based differences and suggest a novel mechanism involved in the differential response of OPCs towards oxidative stress. It conveys a strong message supporting the need to study how complex cellular processes are regulated differently in male and female brains during development and for a better understanding of how the brain copes up with different forms of stress after preterm birth.

## Background

Preterm birth (occurring before 37 weeks of gestation) is a worldwide major health concern due to the long-term neurodevelopmental outcomes in its survivors [1, 2]. The time between 24 and 32 weeks of gestation has been particularly characterized by rapid brain development and is recognized by increased proliferation and migration of neural progenitor cells. The oligodendroglial progenitors are particularly sensitive to injury during this phase. Hence, an insult during this crucial phase leads to a high risk of severe developmental disability including cerebral palsy in these infants [3–5].

Oxygen is considered as a major cause of perinatal insult to the developing brain in preterm infants leading to widespread white matter injury [6–8]. The fact that in most of the cases, preterm infants are born with immature lungs complicates the situation further. They are therefore frequently exposed to increased oxidative stress because of the use of supplemental oxygen often for a long period of time in resuscitation and treatment of neonatal lung diseases [9, 10]. The adverse effects of hyperoxia in preterm infants have been studied widely. A number of studies in cell lines, animal models, and postmortem human brain have revealed that hyperoxia is a powerful trigger for widespread apoptotic neuronal death along with degeneration in the white matter tracts and periventricular region in the developing brain [10–12]. But the molecular mechanisms and details need to be still uncovered.

Interestingly, a number of clinical studies have identified male sex as a well-established, strong independent risk factor for poor neuroanatomical and adverse cognitive outcome following premature birth [13–15]. However, the reasons for their vulnerability are unknown. While rodent models have been developed to study mechanisms of preterm brain injury, few studies have looked at males and females separately [16, 17], limiting the understanding of sex-specific damage and the mechanisms behind sex-related differences. Male susceptibility points towards a possible role of hormones and considering its relation to hyperoxia, it becomes important to identify the underlying reasons for any differential response. Understanding the mechanisms behind such a sex-related difference could eventually pave way to developing better neuroprotective strategies.

In the current study using mouse-derived primary oligodendrocyte progenitor cells (OPCs), we address the molecular background behind the differential response of male- and female-derived OPCs to oxidative stress. We investigated the reasons behind increased vulnerability of male-derived OPCs towards oxidative stress and their inability to give rise to mature oligodendrocytes. We also show how estrogen is involved in mediating a differential outcome in the male and female cells.

## Results

### Hyperoxia leads to impairment of differentiation and disrupts mitochondrial function and stress response pathways in male OPCs

**Fig. 1:**
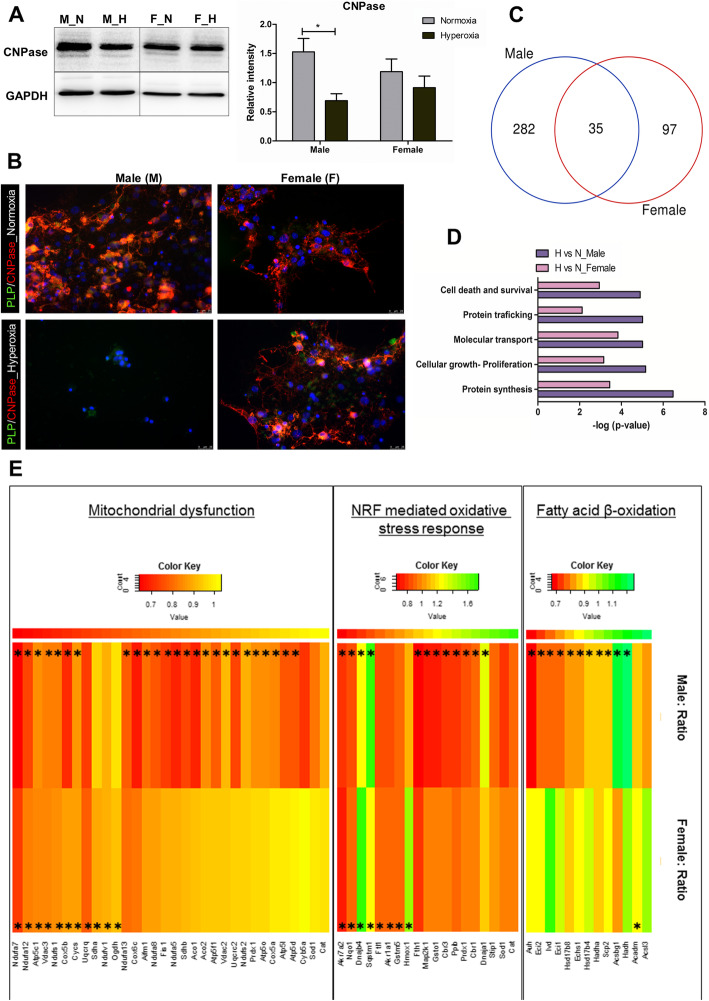
Hyperoxia leads to impairment of maturation and an overall severe effect on the male OPCs. **a** Western blot analysis of male and female OPCs with anti-CNPase antibody at normal (3% O_2_) differentiation conditions and 24 h post 80% O_2_ shock, showing a significant decrease in expression in the male OPCs. In all the figures, M_N represents male normoxia, M_H male hyperoxia, F_N female normoxia, and F_H female hyperoxia. **b** Representative images of male and female OPCs stained for the OPC maturation marker CNPase after differentiation under normal oxygen conditions and post 24 h 80% O_2_ shock. Scale bar represents 75 μm. Data are representative of three independent experiments. Bars and error represent mean ± SEM of replicate measurements. *p < 0.05, **p < 0.01, ***p < 0.001, ****p < 0.0001 (Student’s *t* test). **c** Venn diagram of significantly altered proteins (normalized to the normoxic conditions) in male and female OPCs post 24 h 80% O_2_ treatment. Cutoff *p* value < 0.05. **d** Functional categorization of significantly enriched proteins in male OPCs and female OPCs post 24 h 80% O_2_ treatment using Ingenuity Pathway Analysis (IPA) software. Bar graphs depict the most extensively enriched biological processes among the altered proteins. Cutoff *p* value < 0.05 (Fisher’s exact test). **e** Heat map representation of mitochondrial and stress response proteins that were dysregulated in male- and female-derived OPCs post 24 h 80% O_2_ treatment in comparison to 3% O_2_ (normoxia) controls. Highly downregulated proteins are indicated in red, intermediate in yellow, and highly upregulated proteins in green. Proteins are sorted according to IPA categories. Cells marked with *represents the significantly altered proteins in each group. The cutoff *p* value < 0.07. Data are representative of five independent experiments

We tested different concentrations and time periods of oxygen treatment (corresponding to what is used in the neonatal intensive care) to find an optimum that allowed us to identify the initial target proteins affected by hyperoxia whose effect could be otherwise masked by the later response of large number of proteins responding to widespread severe oxidative stress and cell death. In our model system, we performed experiments with separate male- and female-derived OPCs simultaneously. The cells were given an optimum treatment regime of 80% oxygen shock for 24 h and then were returned back to normal oxygen conditions and were allowed to recover and differentiate for 4 days. At the time of O_2_ shock, the OPCs were largely immature and vulnerable to oxygen damage. The aim of designing the experiment with a recovery time was to see how this exposure of oxygen to the immature cells affects their maturation capability when returned back to normoxia. After this, the protein expression of CNPase was checked in both male and female cells using western blot (Fig. 1a). CNPase was chosen because under the given culture conditions where we did not use any additional specific factors or substrates for inducing advanced myelination, it was relevant to look for differences in the early maturation marker CNPase whose expression is known to gradually increase as maturation progresses. It was found to be significantly less abundant in the male OPCs. Although we observed a difference in the basal level expression of CNPase in both the groups, the effect of oxygen was very prominent on the male cells. A qualitative assessment using immunofluorescence with CNPase antibody showed a much more severe effect of oxygen on the male cells with very few cells even attached to the surface of the plate. On the other hand, the female cells could adhere to the culture plate and also differentiate. Even though the overall observed number of differentiated cells was relatively less as compared to the normoxia control, the female cells looked much more vital and also differentiated much better than the male cells (Fig. 1b). A detailed assessment on the global proteome profile changes using tandem mass spectrometry (MS) revealed a higher number of altered proteins in male than in female cells when compared to the respective normoxic control, with a very little overlap between the identified proteins in both the groups (Fig. 1c). Gene ontology-based cellular function analysis showed that the major cellular functions such as protein synthesis, cellular growth and proliferation, and molecular transport were affected to a much greater extent in the male cells (Fig. 1d). Alterations were also observed in the expression of proteins related to cell adhesion and migration in the male-derived cells post-hyperoxia (Figure S-1). Since the ability of cells to adhere is considered one of the essential factors to initiate cell differentiation, we believe that this could also be an additional factor affecting differentiation post-hyperoxic treatment. Most interestingly, a number of proteins related to mitochondrial function were seen to be significantly downregulated in the male OPCs. Nrf-mediated oxidative stress response pathway and some of the fatty acid oxidation pathway proteins were also seen to be significantly affected in the male cells (Fig. 1e). Giving a very clear picture of the impact of hyperoxia on different molecular functions; these results indicate that the downregulation of these stress response pathways and extensive mitochondrial dysfunction might be critical reasons behind a stronger negative effect on the male cells.

### Oxidative damage in male-derived OPCs is associated with decrease in Nup133 expression

**Fig. 2:**
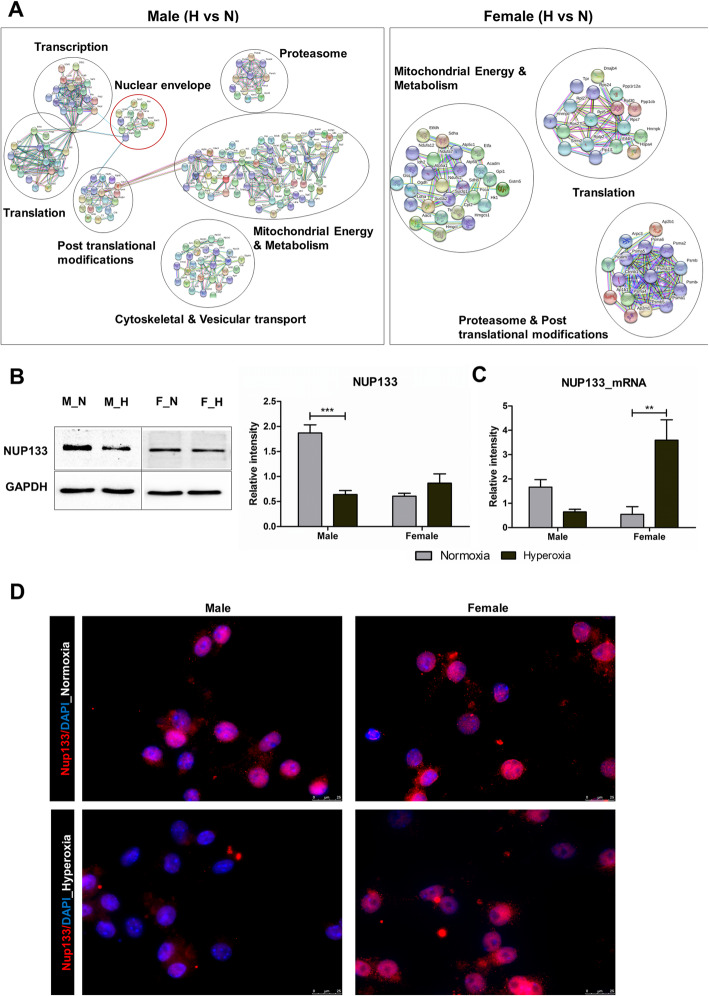
Nuclear envelope (NE) proteins are downregulated in male OPCs as a result of hyperoxia. **a** STRING protein interaction analysis of the significantly altered proteins (*p* < 0.05) in the male (hyperoxia vs normoxia) and female (hyperoxia vs normoxia) groups, with an interaction score of highest confidence (0.900), showing the nuclear envelope proteins to be altered only in the male group. **b** Western blot analysis of male and female OPCs with anti-Nup133 antibody under normal (3% O_2_) conditions and post 24 h 80% O_2_ treatment, showing a significant decrease in expression in the male OPCs. Whereas in female OPCs, Nup133 protein expression showed a trend towards upregulation. ***p < 0.001, **p < 0.01, *p < 0.05 (Student’s *t* test), *n* = 3. Values are means ± SEM. **c** mRNA expression of Nup133 showing downregulation in male OPCs and upregulation in female OPCs post-hyperoxia. ***p < 0.001, **p < 0.01, *p < 0.05 (Student’s *t* test), *n* = 3. Values are means ± SEM. **d** Representative images of mouse male and female OPCs stained for Nup133 after treatment under normal oxygen conditions and post 24 h 80% O_2_ shock. Scale bar represents 25 μm

Protein interaction analysis of the significantly altered proteins as a result of hyperoxia in both male and female cells using STRING interaction networks (Fig. 2a), the independent intensity plots from MS and further validation results (Figure S-2) showed a downregulation of all detected types of lamins and many nucleoporin proteins only in the male-derived cells. Among all these proteins, Nup133 was particularly interesting as it has been shown previously to be crucial for cell differentiation along the neural lineage [18]. It is thought to bind components of chromatin remodeling complexes and repressor/activator complexes with histone-modifying activities in the nucleus to mediate cell differentiation [19]. Independent validations by western blot and qPCR confirmed that Nup133 protein and mRNA levels declined only in the male cells (Fig. 2b–d). Apart from Nup133, the other nuclear envelope proteins analyzed have also been previously reported across different studies to be involved in CNS development [20–23]. Hence, we identify all these nuclear proteins along with Nup133 as novel targets of oxidative stress leading to extended vulnerability in male cells.

Additionally, it was notable that in case of all these nuclear proteins, a basal level difference in expression was observed with a higher abundance in the male cells compared to female cells. A similar difference with a slightly higher abundance in the male cells was also found in case of CNPase (Fig. 1a). This could imply that under similar conditions, the male OPCs differentiated more than the female cells. A previous study in a rodent model [24] supporting this observation reports that at any given time point, the density of oligodendrocytes present in different parts of the male brain are 20–40% higher than in females. Therefore, these results call for deeper understanding of such inherent differences between both the sexes (Figure S-3) which can be helpful to predict cellular behavior.

### Nup133 regulates oligodendrocyte differentiation-related genes and regulates mitochondrial functions through Nrf1

**Fig. 3:**
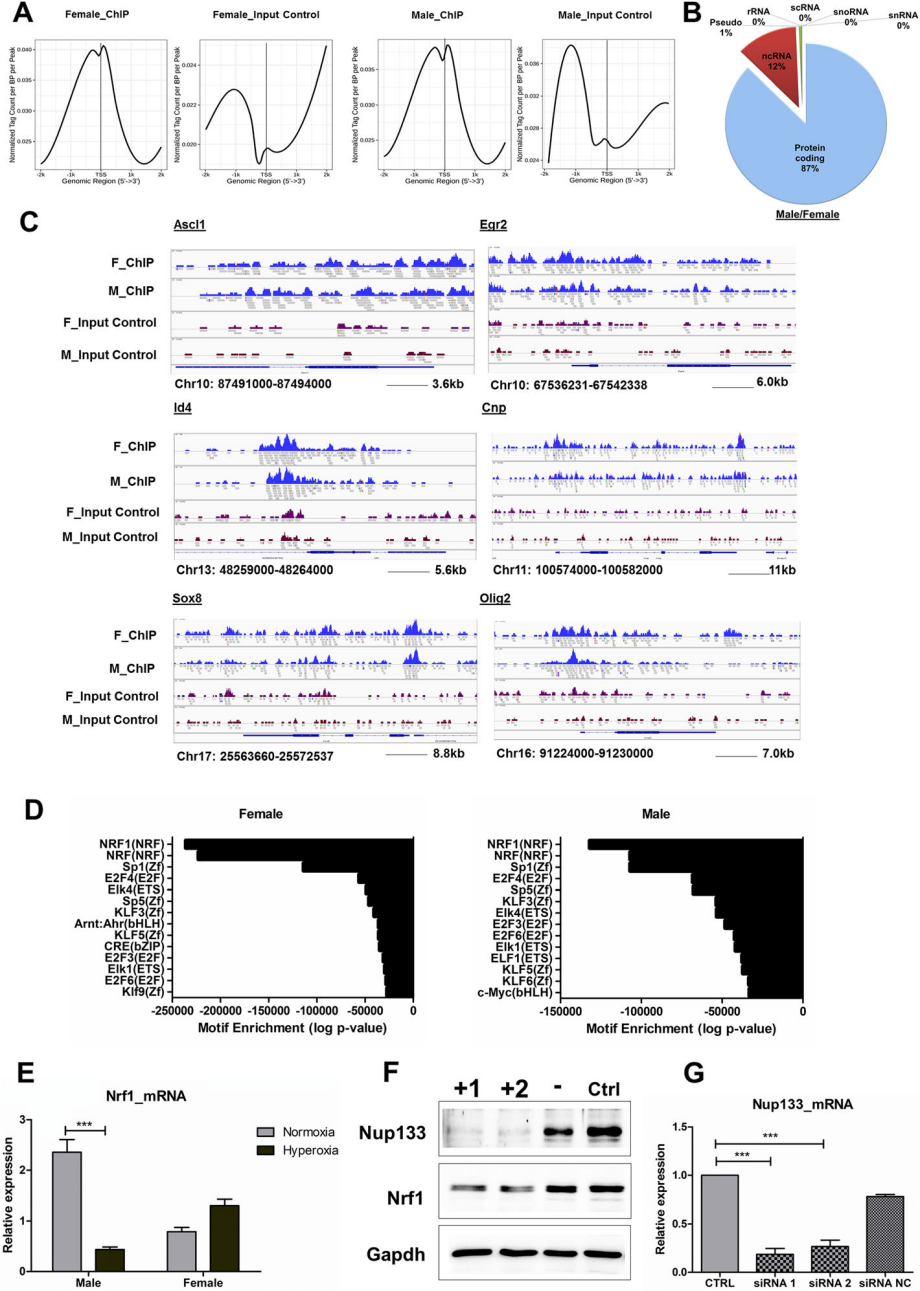
Identification of Nup133 targets in male and female OPCs. **a** Line plot representation of ChIP-Seq signal density for Nup133 male and female ChIP and input controls centered on predicted TSS. **b** Pie chart showing proportions of targets with protein-coding or non-protein-coding designation. **c** Integrative Genomics Viewer (IGV) visualization of Nup133 occupancy at selected target sites related to oligodendrocyte differentiation. ChIP-Seq data are representative of four independent experiments. **d** Known motif analysis of Nup133-binding regions identified by the software HOMER. The highest significant motif was identified as Nrf1 and found to be common in both male and female groups. *q* value determine by Benjamini correction was 0.0000 for all the represented motifs. **e** mRNA expression validation of Nrf1 showing downregulation in male OPCs and a slight upregulation in female OPCs post 24 h hyperoxia. Real-time PCR data is representative of three independent experiments. **f** Western blot of Nup133 and Nrf1 showing +1 for Nup133 specific siRNA 1, +2 for Nup133 specific siRNA 2,—for scrambled siRNA and Ctrl for non-transfected cell lysate. Transfection was performed in the OLN93 cells with 48 h siRNA incubation. Data is representative of three independent experiments. **g** RT-qPCR analysis of Nup133 expression following siRNA transfection for 48 h. Scrambled siRNA (siRNA NC) and untreated sample (Ctrl) are used as controls. Data is representative of three independent experiments

We further chose Nup133 to investigate its downstream targets because even though it is known that Nup133 plays a crucial role in neural cell differentiation, the mechanisms of its action are largely unknown. Assuming that Nup133 binds to the mammalian genome, we performed targeted chromatin immunoprecipitation experiments followed by next generation sequencing to identify its genomic targets. Initially, to evaluate the global distribution of Nup133-occupied regions, we plotted the identified Nup133 bound sites against their distance to the nearest transcription start site (TSS). We detected a strong enrichment for Nup133 occupancy within 1 kb of the nearest annotated TSS in both male and female groups (Fig. 3a), indicating that Nup133 has a stronger occupancy around target promoters. Analysis of targets with protein-coding and non-protein-coding designations showed 87% of all identified targets as protein coding in both male and female cells (Fig. 3b). These results signify that Nup133 apart from its role in transportation might actually be involved in actively regulating gene expression. Most importantly, we found Nup133 occupancy at the promoter sites of many genes known to be critical for oligodendrocyte differentiation, like Cnp, Olig2, Egr2, Sox8, and Id4 (Fig. 3c). We also validated some of these genes by qPCR and the results showed a trend exactly fitting into the hypothesis of Nup133 regulating the expression of these genes (Figure S-4). Hence, we show that Nup133 associates with and is actively involved in modulating the expression of developmentally regulated genes in both male- and female-derived OPCs. Analysis of known motifs using the software HOMER identified nuclear respiratory factor-1 (Nrf1) as the highest ranking significant motif common in both male and female cells (Fig. 3d). Nrf1 is an important transcription factor that activates the expression of various key metabolic genes regulating mitochondrial function and oxidative stress response [25, 26]. This finding strongly advocates for Nrf1 being a direct target of Nup133. To see if a decline in Nup133 protein abundance resulting from hyperoxia in male OPCs could also lead to low abundance of Nrf1, we performed independent qPCR experiments using hyperoxia-treated male and female OPCs. The results showed a matching pattern of Nrf1 expression change post-hyperoxic treatment as was seen in case of Nup133 (Fig. 3e). Nrf1 expression was decreased in male-derived cells post-hyperoxic treatment compared to normoxic control conditions, whereas, in female cells this was not the case. Additionally, small interfering (si) RNA transfection was performed to knock down Nup133 expression in the OLN93 cell line. We found that Nrf1 protein abundance significantly declined upon Nup133 knockdown, proving the regulation of Nrf1 expression in OPCs by Nup133 (Fig. 3f, g). It also explains the drastic effect on mitochondrial and stress response proteins in the male-derived cells that was observed in the proteomic data and sheds light into why the male-derived cells have increased susceptibility towards oxidative damage.

### Nup133 directly interacts with estrogen receptor alpha and together control Nrf1 expression

**Fig. 4:**
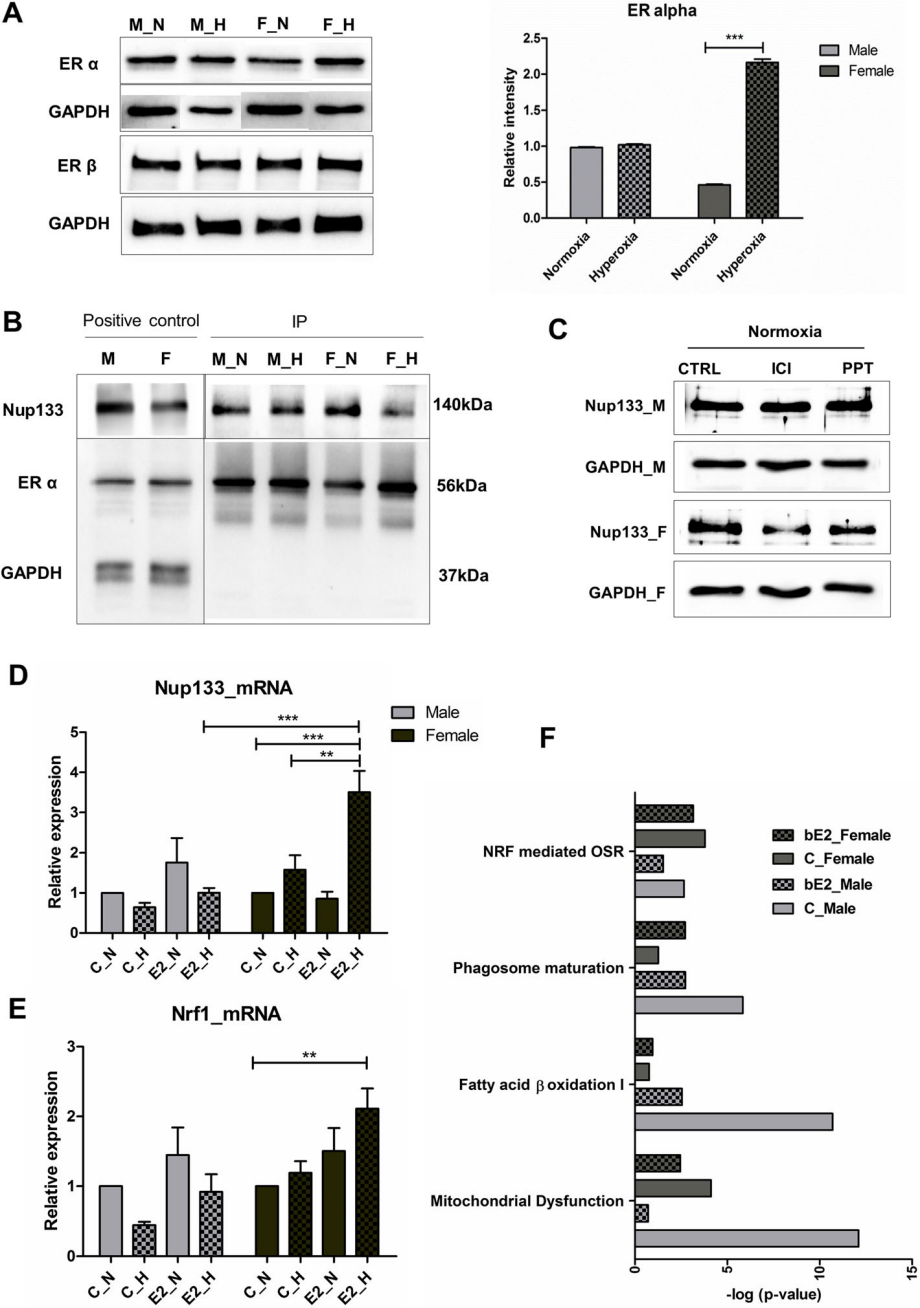
Nup133 directly interacts with ERα. **a** Western blot analysis of male and female OPCs with anti-ERα and anti-ERβ antibodies at normal (3% O_2_) conditions and 24 h post 80%O_2_ shock, showing a significant increase in ERα expression in the female OPCs. **b** Western blot results of co-immunoprecipitation performed using anti-Nup133 antibody showing the interaction between ERα and Nup133 in male and female OPCs under normoxia and hyperoxia. **c** Western blot results showing Nup133 expression post treatment of male and female OPCs with 4,4′,4″-(4-Propyl-[1H]-pyrazole-1,3,5-triyl) trisphenol (PPT) and ICI 182,780 (ICI) under normoxia and 24 h hyperoxia. **d** RT-qPCR results showing Nup133 mRNA expression changes post 17-β estradiol (E2) treatment in male and female OPCs under normoxia and hyperoxia. **e** RT-qPCR results showing Nrf1 mRNA expression changes post 17-β estradiol (E2) treatment in male and female OPCs under normoxia and hyperoxia. **f** Functional categorization of significantly enriched proteins in male and female OPCs post 24 h 80% O_2_ and E2 treatment using IPA. Bar graphs depict the most extensively enriched biological processes among the altered proteins. Cutoff *p* value < 0.05 (Fisher’s exact test). “C” represents control. Data is representative of five independent experiments. All other data are representative of three independent experiments. Bars and error represent mean ± SEM of replicate measurements. *p < 0.05, **p < 0.01, ***p < 0.001, ****p < 0.0001 (Student’s *t* test)

Our experiments with oxygen treatment showed a significant increase in the expression of estrogen receptor alpha (ERα) in the female-derived mouse OPCs indicating a possible role of ERα in oxidative stress response in female cells (Fig. 4a). It was notable that even though the estrogen receptor beta (ERβ) is known to be more prominently expressed in brain tissue [27], we did not observe any notable changes in its expression levels post-hyperoxic treatment in both male- and female-derived cells (Fig. 4a). A number of previous studies have shown that ERα controls Nrf1 expression [28–31]. Hence, to decipher the interaction between Nup133 and ERα in these cells, we performed co-immunoprecipitation experiments using Nup133 antibody and found ERα to be co-precipitated with Nup133, indicating a direct interaction between the two (Fig. 4b). Treatment with the ER antagonist ICI 182,780 (7α,17β-[9-[(4,4,5,5,5-pentafluoropentyl)sulfinyl]nonyl]estra-1,3,5(10)-triene-3,17-diol) decreased Nup133 abundance in the female cells and at the same time treatment with the ERα-specific agonist PPT (1,3,5-tris (4-hydroxyphenyl)-4-propyl-1H-pyrazole) increased Nup133 expression in the female cells under normal conditions, whereas in the male cells, no such effect was observed (Fig. 4c). These results indicate towards the possibility of ERα being an upstream regulator of Nup133 in the female cells. Treatment with 17β-estradiol (E2) showed a significant upregulation of Nup133 and Nrf1 mRNA expression (Fig. 4d, e) in the female cells post-hyperoxia and at the same time, also rendering a positive effect on the male cells post-hyperoxia by bringing back Nup133 and Nrf1 expressions comparable to control normoxia. Proteome analysis of the cells post treatment with E2 under hyperoxia also showed that the mitochondrial dysfunction was significantly reduced in male cells (Fig. 4f). These findings along with the observation that treatment with E2 increased the Nrf1 mRNA levels significantly in female cells post-hyperoxia (Fig. 4e) suggest that steroid hormones and their receptors do contribute to a difference in how Nrf1 is regulated in male and female cells. In female cells, there possibly exists a stronger synergistic regulation via both ERα and Nup133, whereas in the male cells, Nrf1 seems to be regulated mainly by Nup133.

## Discussion

Hyperoxia is well-known to hinder the maturation of OPCs and [9, 32] considering the backdrop of clinical studies mentioning male sex as an independent associated risk factor [14, 17, 33, 34], it was prudent to assume that the male and female brains respond differently to hyperoxic insult and that there might be hormonal factors involved.

Our study shows that there exists a very clear distinction between how the male- and female-derived OPCs respond to high oxygen. We show that treatment with 80% O_2_ for 24 h leads to significant maturation impairment in the male cells as well as increased mitochondrial dysfunction. We identify Nup133 as an important novel target protein influencing these effects in the OPCs. Results of Nup133 targeted ChIP-Seq study revealed its occupancy at the transcription start sites of a number of oligodendrocyte differentiation-related genes, suggesting that it acts as a transcription factor or transcription factor activator/repressor, hence directly regulating the expression of these genes. Alternatively, it may also indirectly induce differentiation by gating the expression of other transcription factors, for instance, Olig1, Egr2, and Hes5. Hence, a downregulation of this important NPC component in the male cells as a result of hyperoxia may lead to disruption of the programmed regulation of the genes required during OPC differentiation. Moreover, the involvement of Nup133 in regulating mitochondrial and oxidative stress response functions through its direct downstream target Nrf1 illustrates that it could be involved in a myriad of overlapping mechanisms not only during cell differentiation but also other important cellular functions. Additionally, its interaction with ERα directs towards this mechanism being linked to hormones. These results therefore point towards the important role of Nup133 in mediating oxidative damage and advocate for further studies on its functions that could provide insight into its role in additional cellular processes and regulatory networks.

Moreover, our finding that hyperoxia leads to alterations in other nuclear envelope proteins in the male-derived cells points towards the severity of the effect on these cells regarding their maturation capability. Like any other cell, differentiation of OPCs into mature oligodendrocytes requires extensive chromatin reorganization and programmed gene repositioning which is achieved by the aid of nuclear envelope components including the nuclear lamina, nuclear membrane, and the nuclear pore complex proteins [35–39]. In the male cells, downregulation of lamin and NPC proteins as a result of hyperoxia disrupts the fine-tuned chromatin reorganization and gene repositioning, resulting in a delay in their maturation. Since the NPCs are the primary channels for communication between the nucleus and the cytoplasm, it is very likely that they have an impact on nearly every cellular process. This could also mean that in a developing brain, such an imbalance of NPC proteins in the OPCs can result in altered cell division capability producing an abnormal pool of OPCs compromised on their ability to give rise to mature oligodendrocytes. Thereby, these facts might explain why the brain fails to recover after hyperoxic injury even though it is known that there is an apparent replenishment of OPCs [40, 41]. Their subsequent failure to differentiate might lead to long-term effects in the male subjects. Hence, this calls for a more detailed study into the compositional changes in the NPC and the functional significance of these variations in brain development could be useful for understanding the underlying pathologies of various developmental issues. This will be particularly beneficial for fully understanding the role of NPC components in various disease models.

Steroid hormones have been long implicated in neuroprotection. However, the exact mechanisms how these hormones act on the cells to rescue them under conditions of stress is yet to be completely deciphered. Our results show that the classical estrogen receptor alpha (ERα) acts as a possible upstream regulator of Nup133 and that there exists a difference in the extent of regulation via ERα in the male and female cells with a stronger synergistic regulation in the female cells allowing them to better cope up with oxidative stress. By means of this mechanism, we have explained how E2 can have a differential effect on the male and female cells. Addition of estradiol protects the cells from oxidative stress as reflected by the changes in Nup133 and Nrf1 mRNA levels.

## Conclusions

Our experimental results show that CNPase protein levels decline following hyperoxia in male but not in female cells. Hence leading to inhibition of maturation in male cells as an effect of high oxygen.

We show that Nup133 protein levels decline following hyperoxia in male but not in female cells and its mRNA level is increased following hyperoxia in female but not in the male cells. We identify Nrf1 as a target of Nup133 and show that Nrf1 mRNA declines following hyperoxia in male but not in female cells. Additionally, ERα is upregulated following hyperoxia in female but not in male cells.

These results along with the drastic changes observed in mitochondrial and stress response proteins strongly indicate the need to study cellular responses in both the sexes separately. Regardless of the cell type investigated or the type of insult, the fact that male and female cells can respond differently makes this study important. We believe that the presence of inherent differences between the sexes should not be ignored while decoding any cellular mechanism.

To conclude, we have uncovered molecular alterations being associated with a differential hyperoxia response in male and female OPCs which might contribute to the susceptibility of male OPCs towards oxidative stress. At the same time, we showed that an additional synergistic regulation via ERα may render resistance to the female cells against oxidative stress. At a functional level, our findings on Nup133 playing a key role in regulating cell differentiation as well as cellular stress response provide insight into a probable new mechanism of hyperoxia-induced damage in OPCs and thereby might point to a new function of Nup133, opening up new avenues for understanding the molecular mechanisms behind hyperoxia-induced white matter damage in the developing brain. The involvement of Nup133 in mediating such a response has broad implications because in humans, Nup133 is a constitutive cellular protein expressed in a lot of different cell types including a number of cancers. Other than being just a transporter protein, its role in other cellular processes still needs to be studied in detail.

By means of this study, we reveal the importance of detailed investigations into sex-based differences during fetal development and the role of nucleoporin proteins not only in normal brain development but also under various stress/disease conditions. We believe that changes in these essential cell components could be linked to the reasons behind some of the observed long-term neurological effects in preterm infants.

## Methods

### Animals

Animal colonies were housed and maintained following the international FELASA, national GVsolas, and local University of Greifswald animal research guidelines. The PLP-GFP transgenic mouse line in which expression of the GFP reporter gene is driven by the Plp regulatory sequences in a C57BL/6 J background mouse strain was obtained as a kind gift from Prof. Bernard Zalc (Centre de Recherche de l’Institut du Cerveau et de la Moelle epiniere UPMC-Paris6, UMR_S 975; Inserm U 975; CNRS UMR 7225, Paris) [42]. Transgenic mice did not display any obvious developmental defects. Crl:CD-1 (ICR) mice were received from Charles River Company and breed for maximal two generations at the facility to get the donor mice. Both strains were used to generate oligodendrocyte precursor cells.

### Isolation and culture of mouse OPCs

Mouse OPCs were isolated from enzymatically dissociated P2-P4 (post-natal day 2–4) old mice brains. For each isolation, tissue from the midbrain region comprising the sub-ventricular zone from 3 male and 3 female (from the same litter) brains was pooled separately. The sex of the pups was determined visually and with genotyping. All animal usage for cell isolation was performed according to the institutional regulations regarding animal ethics.

Isolated cells were cultured in serum free-growth medium supplemented with B-27(w/o retinol), EGF 0.02 μg/ml, and FGF 0.005 μg/ml under 3% O_2_ conditions. After ~ 5–6 days when the neurospheres were around 100 μm in diameter, gradually the neurosphere growth medium was replaced by serum-free B104CM (conditioned medium prepared from B104 neuroblastoma cell line) containing oligosphere medium every alternate day for 2 weeks. After this time period, the cells were trypsinized and used for experiments. Detailed protocol for the isolation and culture of mouse OPCs has been published [43].

We used 3% O_2_ as the normoxic condition to cultivate these cells as hypoxia is particularly important for the central nervous system, where oxygen (O_2_) levels range from 8% at the pia to 0.5% in the midbrain. These cells were isolated from the midbrain region. Hence, it was relevant to cultivate them at 3% O_2_ since the traditional in vitro stem cell systems use oxygen tensions that are far removed from the in vivo situation (21% O_2_) [44–49].

### Treatment with hyperoxia

For differentiation experiments, the male- and female-derived OPCs were trypsinized separately, and the cells were counted and seeded in equal densities into 6-well plates for adherent cells (without any coating; Cell+, Sarstedt, Germany) containing 1 ml oligosphere medium per well. The cells were first given 80% O_2_ shock for 24 h, after which, 1 ml fresh media was added to each well and all plates were returned to 3% O_2_ (normoxia) condition. The cells were then allowed to differentiate for 4 days with 1 ml medium change every alternate day.

For protein and RNA studies, the cells from male- and female-derived OPCs were trypsinized, counted, and seeded in equal densities into 6-well plates for adherent cells (without any coating; Cell+, Sarstedt) containing 1 ml DMEM/F12 + 45% glucose solution (666 μl for 50 ml DMEM/F12) medium per well. The plates were then kept at 80% O_2_, 5% CO_2_ and 37 °C (hyperoxia), and 3% O_2_, 5% CO_2_, and 37 °C (normoxia) incubators respectively, for 24 h. Cells were subjected to protein and RNA extraction immediately after the treatment time. All cells (adhered and suspended) from each well were used for further processing and analysis.

### Treatment with E2, ICI and PPT

For differentiation experiments as well as for protein and RNA studies, the procedure was followed exactly as mentioned above but with the addition of 100 nM E2, 1 µM ICI or 100 nM PPT (final concentration) before giving the oxygen shock.

### Cell lines

The OLN93 cell line was obtained from Prof. Dr. Christiane Richter-Landsberg (Universität Oldenburg, Germany). It is a rat derived (female), pre-OL adherent cell line derived from spontaneously transformed cells in primary rat brain glial cultures [50]. The cell line was authenticated by the Leibniz Institute DSMZ-German Collection of Microorganisms and Cell Cultures, Braunschweig, Germany.

The cells were cultured as per the protocol mentioned by Gerstner et al. [51] in Dulbecco’s Modified Eagle’s Medium (DMEM) (with 3.7 g/l NaHCO_3_, 25 mM HEPES, 4.5 g/l D-Glucose, 4.4 g/l NaCl; Biochrom), supplemented with 10% heat inactivated fetal calf serum (FCS, Biochrom), 0.01% human serum albumin (HSA, Grifols), and 1% penicillin-streptomycin solution. Cultures were kept in a 37 °C, 5% CO_2_ incubator, and media was exchanged every 2–3 days.

B104 neuroblastoma cell line was purchased from the American Type Culture Collection (ATCC). The cells were used only for preparing conditioned media for OPC culture as described previously [43].

### Preparation of protein extracts and mass spectrometric analysis

Proteins were extracted in 8 M urea/2 M thiourea and subjected to tryptic digestion. Peptides were analyzed by LC-ESI tandem mass spectrometry on a LTQ-Velos Orbitrap mass spectrometer (Thermo Scientific, Bremen, Germany). Qualitative and quantitative analyses of mass spectra were performed by the Rosetta Elucidator software (Ceiba Solutions, Boston, MA, USA) and statistical analysis with Analyst (Genedata, Basel, Switzerland). Detailed information is provided in supplementary experimental procedures.

### Immunoblot analysis

Protein extracts from whole cell lysates containing ∼ 40 μg protein were loaded in each lane of a Mini-Gel module for electrophoresis (BioRad, Munich, Germany). Protein was transferred onto nitrocellulose membrane (Amersham Protran 0.45 μm NC Western Blotting Membrane, GE Healthcare, USA), blocked with 1x Blocking buffer (Pierce™ Protein-Free (TBS) Blocking Buffer, Thermo Fisher) at RT for 1 h, and incubated in primary antibody at 4 °C overnight. GAPDH (Rabbit Anti-GAPDH (D16H11) mAb, Cell Signaling Technology) was used as the loading control at 1:1000 dilution. Blots were incubated with secondary antibody at 1:10000 dilution in blocking buffer for 1 h at RT. Protein bands were visualized with SuperSignal™ West Femto Maximum Sensitivity Chemiluminescence Substrate (Thermo Scientific). Densitometric intensities were calculated using the ImageLab software (BioRad). Primary antibodies are listed in supplementary experimental procedures.

### Quantitative reverse-transcription polymerase chain reaction

Total RNA from pelleted cells was isolated using TRIzol reagent (Life Technologies, Germany). First-strand cDNA synthesis was performed using the QuantiTect Reverse Transcription Kit from QIAGEN (Hilden, Germany). The prepared cDNA was used as a template for quantitative PCR reactions using primer-probes designed against specified mRNA transcripts (NCBI Primer Design tool). Reactions were performed using PowerUp™ SYBR™ Green Master Mix (Thermo Scientific). Ct values were calculated using automatically determined threshold values using the Step One Plus software (Applied Biosystems, Darmstadt, Germany).

### Immunofluorescence

For immunofluorescence staining (IF), cells were fixed in 4% paraformaldehyde, rinsed twice in 1x PBS and incubated at room temperature in a blocking solution (3% normal goat serum in 1x PBS + 0.3% Triton x100), followed by incubation with primary antibodies at 4 °C overnight. Samples were then washed 3 times with phosphate buffered saline-Tween 20 (PBST) and incubated with fluorescence conjugated secondary Alexa antibodies (Life Technologies) at room temperature for 2 h. Slides were mounted with Vectashield Mounting Medium with Dapi (Vector Laboratories, UK) and imaged on Leica SP5 microscope (Leica, Germany).

We checked for cell death using Propidium Iodide staining/uptake (microscopic analysis) in each of the immunofluoresence experiments after 24 h 80% O_2_ treatment (from the time of seeding into 8-well chamber slides) and found that hardly approximately 2% of the floating cells were stained PI positive in the male OPCs, while in females, it was even lesser. But due to difficulty in quantifying the floating cells, the data is not mentioned. Even representative images from different planes could not show any considerably visible PI stained cells.

However, we have proteomic data supporting this observation that after 24 h 80% O_2_ treatment, cell adhesion-related proteins are downregulated in the male population which has been mentioned in the supplementary information. Additionally, the proteome analysis also revealed that apoptosis or cell death-related proteins were not upregulated significantly in both the populations, ruling out widespread cell death.

### Chromatin immunoprecipitation (ChIP)

Male- and female-derived OPCs without any treatment were subjected to ChIP using anti-rabbit NUP133 polyclonal antibody (Proteintech, UK) at a concentration of 10 μg per reaction. Chromatin immunoprecipitation was performed using the MAGnify^TM^ Chromatin Immunoprecipitation System kit (Thermo Fisher). The manufacturers’ protocol provided with the kit was followed to perform the experiments.

### ChIP-Illumina NextGen sequencing and data processing

The prepared ChIP output DNA from 4 individual bio-replicates, along with an input control for each sample was used to prepare Illumina TruSeq nano ChIP sequencing libraries. The Illumina NextSeq 500 platform and a high-output v2 1 × 75 bp cycle kit were used to sequence the Illumina TruSeq ChIP libraries. The produced single-end reads which passed Illumina’s chastity filter were subjected to de-multiplexing and trimming of Illumina adaptor residues using the Illumina’s bcl2fastq software (version v2.19.1.403). The quality of the reads was checked with the software FastQC (version 0.11.7). Subsequently, the reads were mapped to the mm10 reference genome using the software BWA-MEM (version 0.7.17). PCR and optical read duplicates were removed from the mapping files using the software Picard (version 2.9.0). Peak calling, annotation (e.g., distance to nearest transcription starting site), motif search and initial pathway analysis were done with the software HOMER (version 4.9). The Integrative Genomics Viewer (IGV) software was used for further analysis.

### Transfection of OLN93 cells

Transfection experiments were performed using Lipofectamine RNAiMAX Transfection Reagent (13778-150, Thermo Fisher Scientific) according to the manufacturer’s protocol. Reverse transfection was the chosen method used for performing the experiments. For each experiment, cultures of OLN93 cells (passages 25–32) were trypsinized, counted (2 × 10^6^), and seeded into standard 6-well plates into which the siRNA-lipofectamine complexes were directly added at the same time. A final siRNA concentration of 50 pm per well was used. Stealth RNAi™ siRNA Negative Control, Med GC (12935300, Thermo Fisher Scientific) was used as the negative control. Cells were incubated at 37 °C, 5% CO_2,_ and normal room air for 48 h. They were then subjected to RNA (with Trizol) and protein isolation.

### Experimental design and statistical analysis

All qPCR and Western blot data analysis were performed using the one-way ANOVA between groups followed by Tukey HSD post hoc *t* tests for multiple comparisons. Other statistical parameters including the exact value of *n*, the definition of center, dispersion and precision measures (mean ± SEM), and statistical significance are reported in the figures and figure legends. Data was judged to be statistically significant when *p* < 0.05, if not otherwise indicated.

## Supplementary information

**Additional file 1.** Supplemental Experimental Procedures

**Additional file 2: Figure S1.** Proteins involved in cell adhesion and migration downregulated in male OPCs. (A) Immunoblot analysis of RhoA protein showing downregulation in male OPCs post 24 h 80%O_2_ treatment. (B) Heat-map representation of cell adhesion related proteins that were dysregulated in male and female derived OPCs post 24 h 80%O_2_ treatment in comparison to 3%O_2_ (normoxia) controls. Mapped expression ratios are depicted with a color scale as shown in the figure, such that highly downregulated proteins are indicated in red, intermediate in yellow, and highly upregulated proteins in green. Proteins are sorted according to Gene ontology (biological process). Dark outlined cells represent the significant proteins in each group. The cut off p value being 0.07. Data are representative of five independent experiments. (C) Independent intensities of Rac1, Mapk1, Map 2k1 and Arpc1b plotted from. MS results showing a significant downregulation in male derived-OPCs post hyperoxia. Data are representative of three experiments. Bars and error represent mean ± SEM of replicate measurements. ∗p < 0.05, ∗∗p < 0.01, ∗∗∗p < 0.001 (Student’s t test). **Figure S2.** Changes in nuclear envelope proteins in OPCs post hyperoxia. (A) Western blot analysis of male and female OPCs with anti-Nup-50 and anti-Lamin B1 antibodies under normal (3%O_2_) conditions and post 24 h 80%O_2_ treatment, showing a significant decrease in expression in the male OPCs. Whereas in female OPCs, Nup50 showed a significant upregulation post hyperoxia. ***p < 0.001, **p < 0.01, *p < 0.05 (Student’s t test), n = 3. Values are means ± SEM. (B) mRNA expression of Nup210 showing downregulation in male OPCs and upregulation in female OPCs post hyperoxia. ***p < 0.001, **p < 0.01, *p < 0.05 (Student’s t test), n = 3. Values are means ± SEM. (C) Intensities of Lamin B1, Lamin B2, Pre-Lamin A/C, Nup210, Nup155 and Nup98 plotted from the mass spectrometry results show a significant downregulation of Lamin B1, Lamin B2, Nup155 and Prelamin A/C in male derived OPCs post hyperoxia. For Nup210 and Nup98 a similar trend in both cell groups was observed. ∗p < 0.05, ∗∗p < 0.01, ∗∗∗p < 0.001, ∗∗∗∗p < 0.0001 (Student’s t test), n = 3. Values are means ± SEM. **Figure S3.** Inherent protein profile differences between male and female OPCs under normal conditions. (A) Functional categorization of significantly different proteins in male and female OPCs under normal conditions using IPA. Bar graphs depict the most extensively enriched biological processes among the significantly different proteins. Cut off p value being 0.05. (B) STRING Protein interaction analysis of the proteins detected to be significantly different (*p* < 0.05) in the male vs female (normoxia) group from the MS data, with an interaction score of high confidence (0.700), showing proteins related to energy and metabolism, protein synthesis and a few cytoskeletal proteins to be differentially expressed in male and female cells. **Figure S4.** Nup133 ChIP-Seq additional results and validation. (A) Pie chart showing proportions of genomic landmarks corresponding to Nup133-bound targets. (B) De novo motif analysis of Nup133-binding regions identified different motifs with the highest significance ranking in male and female groups. The q-values for all the presented motifs were determined as 0.01. ChIP-Seq data are representative of four independent experiments. (C) mRNA expression validation of Cnp and Egr2 showing downregulation in male OPCs post hyperoxia. Whereas Hes 5 that acts as a transcription repressor and a negative regulator of oligodendrocyte differentiation is upregulated in male OPCs post hyperoxia. Data are representative of three independent experiments. Bars and error represent mean ± SEM of replicate measurements. ∗p < 0.05, ∗∗p < 0.01, ∗∗∗p < 0.001, ∗∗∗∗p < 0.0001 (Student’s t test). **Figure S5.** Validation of Nup133 antibody and ChIP-Seq targets by ChIP-qPCR. (A) Nup133 immunoprecipitation (IP) detected by immunoblot in male and female derived OPCs without any treatment. Positive control shown here is cell lysate obtained from male OPCs and negative control is the pull down product obtained from IP performed using control IgG antibody. (B) Western blot detection of Nup133 bands in cell lysates prepared from untreated OPCs. (C) Representative immunofluorescence images of OPCs stained for Nup133 at different focal plains. Scale bar represents 25 μm. (D) IGV genome browser tracks showing enrichment peaks for each respective target. Primers were designed specifically ON-target covering the highest peak areas. Graphs depicting enrichment over total genomic input (%Input) for male, female and mock IgG (IgG) control conditions for 6 chosen gene targets identified by ChIP-Seq. Results for all ChIP-qPCR data were generated from three independent experiments.

## Abbreviations

CNPase: 2′,3′-cyclic-nucleotide 3′-phosphodiesterase
CNS: Central nervous system
TSS: Transcription start site
E2: 17β-estradiol
ER: Estrogen receptor
EGF: Epidermal growth factor
FGF: Fibroblast growth factor
Nrf1: Nuclear respiratory factor 1
MS: Mass spectrometry
GAPDH: Glyceraldehyde 3-phosphate dehydrogenase
ICI: ICI 182,780 (fulvestrant)
PPT: (1,3,5-tris (4-hydroxyphenyl)-4-propyl-1H-pyrazole)
OPC: Oligodendrocyte precursor cell
