# Supplementary material for: Nup133 and ERα mediate the differential effects of hyperoxia-induced damage in male and female OPCs
Source: Mol Cell Pediatr. 2020 Aug 25;7:10. doi: 10.1186/s40348-020-00102-8 (PMC7447710; doi:10.1186/s40348-020-00102-8)
Supplement: Supplementary file 1 — Additional file 1. Supplemental Experimental Procedures [file 40348_2020_102_MOESM1_ESM.docx]

# SUPPLEMENTAL EXPERIMENTAL PROCEDURES

**Mass spectrometry**

Cells from five biological replicates were harvested in 8M urea/2M thiourea and proteins extracted by 5 cycles with 10 min incubations at 37^o^C at 1400 rpm and freezing in liquid nitrogen for 5 min. Nucleic acids were fragmented by sonification and insoluble cell compartments pelleted via centrifugation (1 h, 15.600 g, 20°C). The supernatant was stored at -80 °C. Bradford protein assay was used to determine the protein concentration. A volume equivalent to 4 μg of protein was filled up to a total volume of 20 μL with 20 mM ammonium bicarbonate (ABC), 1 μL DTT (25 mM) was added and samples were incubated at 60 °C for 1 h. Seven μL ABC were added and released thiol groups were alkylated with 2 μL 100 mM iodoacetamide (15°min at 37 °C in darkness). Samples were digested with trypsin in a ratio of 1: 50 (protease: protein) over night at 37°C. The digestion was stopped via addition of acetic acid in a final concentration of 1%. Resulting peptide extracts were purified at C18 material (ZipTip, Merck Millipore, Darmstadt, Germany).

Analyses were carried out on an LC-coupled ESI-tandem mass spectrometer as described earlier ([Murr et al., 2017](#_ENREF_1)). Separation of peptides was achieved on a reverse phase nano-Acquity UPLC column (1.7 μm, 100 μm i.d. × 100 mm, Waters GmbH, Eschborn, Germany) using a 90 min non-linear gradient ranging from 2 to 60% acetonitrile in 0.1% acetic acid at a flow rate of 400 nl/min. The detection was carried out with a LTQ-Orbitrap-Velos mass spectrometer (Thermo Electron, Bremen, Germany) in data-dependent mode selecting 20 precursor ions with the highest intensities for CID fragmentation per cycle. Once measured masses were excluded 60 s from a new fragmentation. Proteins were identified by searching in a forward-reverse UniProt/SwissProt database (rel. 2014_01) with a restriction on *Mus musculus* and using the SorcererTM software platform with Sequest algorithm implemented in Rosetta Elucidator software suite (Ceiba Solutions, Boston, MA, USA). A statistical evaluation of the results was performed by analysis in peptide/protein prophet and the annotation of peptides was carried out at a false-positive rate of <1% that is equivalent to a peptide probability >0.87. Only proteins with a probability >0.8 and a peptide count ≥2 were used for statistical analyses carried out in Analyst (Genedata, Basel, Switzerland). For functional categorization of differentially abundant proteins (p-value<0.05) Ingenuity Pathway analysis, STRING and Go annotation as provided by UNIPROT database were used.

**Immunoblot Analysis and Immunofluorescence**

Primary antibodies used are listed below.

| **REAGENT or RESOURCE** | **SOURCE** | **IDENTIFIER** |
| --- | --- | --- |
| **Antibodies** | | |

| Rabbit polyclonal anti- NUP133 | Proteintech Group | Cat# 12405-1-AP; RRID:AB_2154450 |
| --- | --- | --- |
| Rabbit polyclonal anti- NUP50 | Novus | Cat# NB100-93324; RRID:AB_1237257 |
| Rabbit monoclonal anti-lamin B1 (clone D9V6H) | Cell Signaling Technology | Cat# 13435; RRID:AB_2737428 |
| Mouse monoclonal anti-CNPase (clone 11-5B) | Abcam | Cat# ab6319; RRID:AB_2082593 |
| Goat Anti-Mouse IgG (H+L) Alexa Fluor 594 Conjugate | Thermo Fischer Scientific | Cat# A-11005;  RRID:AB_2534073 |
| Anti-rabbit IgG, HRP-linked Antibody | Cell Signaling Technology | Cat# 7074P2;  RRID:AB_2099233 |
| Rabbit Anti-GAPDH (D16H11) mAb | Cell Signaling Technology | Cat# 5174;  RRID:AB_10622025 |
| Goat Anti-Rabbit IgG (H+L) Alexa Fluor 594 Conjugate | Thermo Fischer Scientific | Cat# A-11012;  RRID:AB_2534079 |
| Peroxidase-AffiniPure F(ab')2 Fragment Goat Anti-Mouse IgG (H+L) | Jackson ImmunoResearch Labs | Cat# 115-036-146;  RRID:AB_2307347 |

| RhoA (67B9) Rabbit mAb antibody | Cell Signaling Technology | Cat# 2117; RRID:AB_10693922 |
| --- | --- | --- |

**Quantitative Reverse-Transcription Polymerase Chain Reaction**

# List of primer sequences used for PCR.

The primers were designed as mentioned in the methods part using NCBI primer design tool. ‘F’ stands for forward primer and ‘R’ for reverse primer. Sry (sex determining region Y) primers were used for genotyping.

| **Primer** | **Sequence (5’-> 3’)** |
| --- | --- |
| SRY_Mouse_F | GTGAGAGGCACAAGTTGGC |
| SRY_Mouse_R | TCTTAAACTCTGAAGAAGAGAC |
| Cnp_F_ChIP | GGAGGGTGGTAGGTACCAAGA |
| Cnp_R_ChIP | GCTTTGCCAGAGCAGGAGAT |
| Egr2_F_ChIP | CTCGTCGGTGACCATCTTCC |
| Egr2_R_ChIP | GAAGACACGCGGCTTACCTC |
| Nrf1_F_ChIP | CTGCAGGTCCTGTGGGAAT |
| Nrf1_R_ChIP | CATCCAACGTGGCTCTGAGT |
| Fam187a_F_ChIP | GGACGTTTCTGCAGTGGTCT |
| Fam187a_R_ChIP | AACCGCCCATCAAAGTCTGT |
| Hes5_F_ChIP | TAATCGCCTCCAGAGCTCCA |
| Hes5_R_ChIP | GCGAGTTGCACTTACTCGGT |
| Olig1_F_ChIP | TGCGCGAAGTTATCCTACCC |
| Olig1_R_ChIP | GCAATCTTGGAGAGCTTGCG |
| Sirt2_F_ChIP | GAGGGTGGTGAGGTAGGGAA |
| Sirt2_R_ChIP | TGCTCCCAGTCCCCATACTA |
| Gapdh_F | GTGTTCCTACCCCCAATGTGT |
| Gapdh_R | ATTGTCATACCAGGAAATGAGCTT |
| Hprt1_Ms_F | CCTAAGATGAGCGCAAGTTGAA |
| Hprt1_Ms_R | CCACAGGACTAGAACACCTGCTAA |
| Nup210_F | GCCTCCTGTCACACTTCCTG |
| Nup210_R | TGAAAGCAGCCGGAGATGAG |
| Nup133_F | GTTTCCGAGCGTCTCCTCTC |
| Nup133_R | CGAGTTAACCGCAGATCCCA |

**ChIP-qPCR (NGS Validation)**

Validation of the NGS results was performed using ChIP-qPCR. Three independent ChIP experiments were performed using exactly the same protocol described above for ChIP-Seq and the data was generated from separate qPCR runs. The identified peaks of the genes of interest were overlaid on the reference genome using the USCS genome browser to identify the specific regions of the target genes to which the detected peak sequence aligned. Primers were then designed using these specific regions of the gene to yield a maximum product size of not more than 100bp. The NCBI Primer design tool, primer-BLAST (https://www.ncbi.nlm.nih.gov/tools/primer-blast/) was used to design the primers using RefSeq mRNA for reference to flank each targeted peak site. Primer pairs were cross-checked for spurious hybridization potential using UCSC In-Silico PCR web server (https://genome.ucsc.edu/cgi-bin/hgPcr?db=mm10) against the mouse (mm10) reference genome to minimize spurious hybridization. The used primer sequences are listed in the table above.

**Transfection of OLN93 cells**

Details of siRNA used are as listed below:

| **Si RNA sequence** | | |
| --- | --- | --- |
| siRNA sequence (5’-3’) : Nup133 #1:  GAUUGCUUCCUGGACAGCUACGUUU  AAACGUAGCUGUCCAGGAAGCAGUC | Thermo Fischer Scientific | Cat # 1330001;  Assay ID# RSS307530 |
| siRNA sequence (5’-3’) : Nup133 #2:  GCACCACAGCUCAUCAGUCUGUAUA  UAUACAGACUGAUGAGCUGUGGUGC | Thermo Fischer Scientific | Cat # 1330001;  Assay ID# RSS307532 |

**References**

Murr, A., Pink, C., Hammer, E., Michalik, S., Dhople, V.M., Holtfreter, B., Volker, U., Kocher, T., and Gesell Salazar, M. (2017). Cross-Sectional Association of Salivary Proteins with Age, Sex, Body Mass Index, Smoking, and Education. Journal of proteome research *16*, 2273-2281.
