# Supplementary figures and images for: Nup133 and ERα mediate the differential effects of hyperoxia-induced damage in male and female OPCs
Source: Mol Cell Pediatr. 2020 Aug 25;7:10. doi: 10.1186/s40348-020-00102-8 (PMC7447710; doi:10.1186/s40348-020-00102-8)

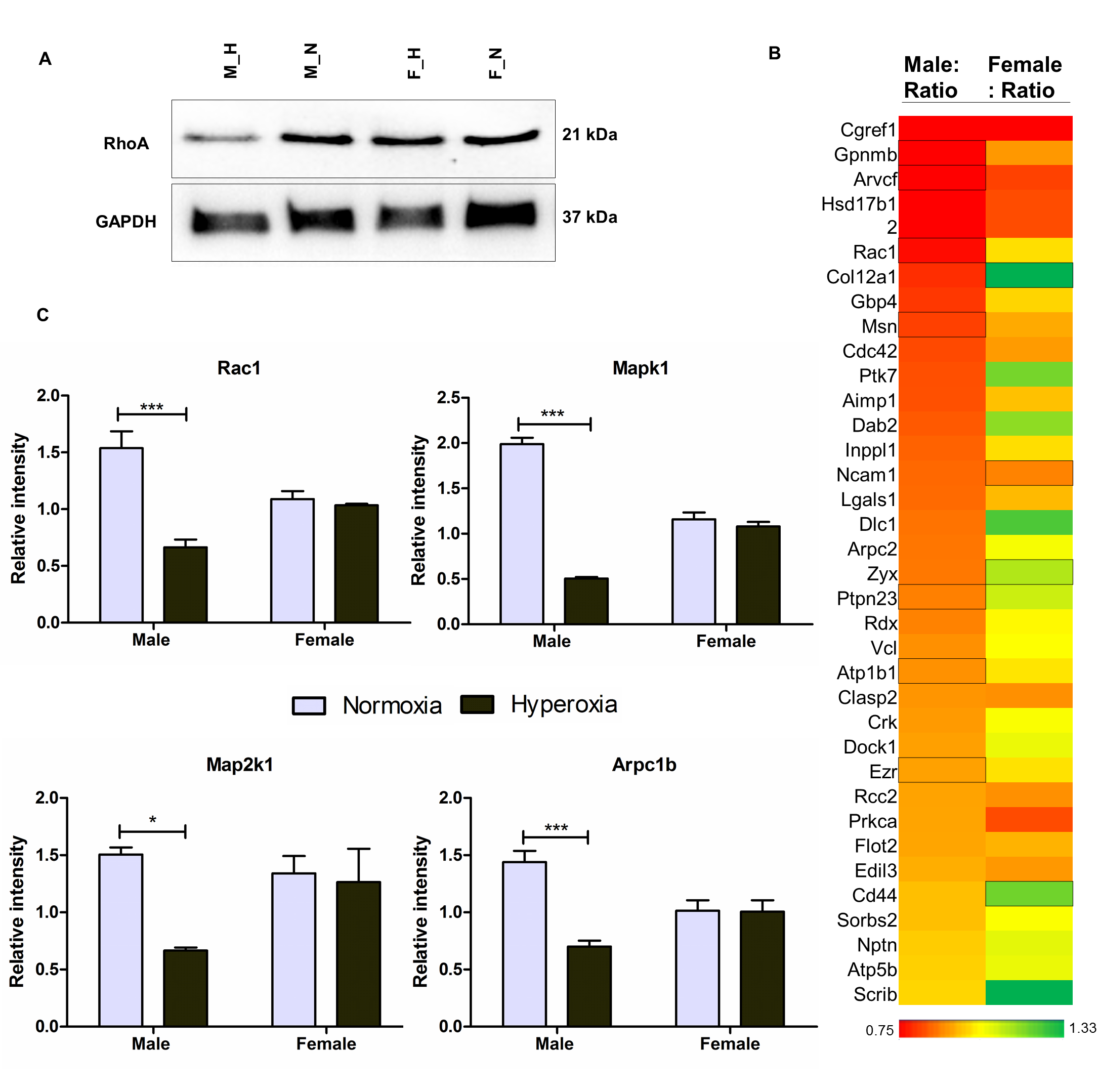

Supplement: Supplementary file 2 — Additional file 2: Figure S1. Proteins involved in cell adhesion and migration downregulated in male OPCs. (A) Immunoblot analysis of RhoA protein showing downregulation in male OPCs post 24 h 80%O2 treatment. (B) Heat-map representation of cell adhesion related proteins that were dysregulated in male and female derived OPCs post 24 h 80%O2 treatment in comparison to 3%O2 (normoxia) controls. Mapped expression ratios are depicted with a color scale as shown in the figure, such that highly downregulated proteins are indicated in red, intermediate in yellow, and highly upregulated proteins in green. Proteins are sorted according to Gene ontology (biological process). Dark outlined cells represent the significant proteins in each group. The cut off p value being 0.07. Data are representative of five independent experiments. (C) Independent intensities of Rac1, Mapk1, Map 2k1 and Arpc1b plotted from. MS results showing a significant downregulation in male derived-OPCs post hyperoxia. Data are representative of three experiments. Bars and error represent mean ± SEM of replicate measurements. ∗p < 0.05, ∗∗p < 0.01, ∗∗∗p < 0.001 (Student’s t test). Figure S2. Changes in nuclear envelope proteins in OPCs post hyperoxia. (A) Western blot analysis of male and female OPCs with anti-Nup-50 and anti-Lamin B1 antibodies under normal (3%O2) conditions and post 24 h 80%O2 treatment, showing a significant decrease in expression in the male OPCs. Whereas in female OPCs, Nup50 showed a significant upregulation post hyperoxia. ***p < 0.001, **p < 0.01, *p < 0.05 (Student’s t test), n = 3. Values are means ± SEM. (B) mRNA expression of Nup210 showing downregulation in male OPCs and upregulation in female OPCs post hyperoxia. ***p < 0.001, **p < 0.01, *p < 0.05 (Student’s t test), n = 3. Values are means ± SEM. (C) Intensities of Lamin B1, Lamin B2, Pre-Lamin A/C, Nup210, Nup155 and Nup98 plotted from the mass spectrometry results show a significant downregulation of Lamin B1, Lamin B2, [file 40348_2020_102_MOESM2_ESM.zip › Supplementary Figure S-1.tif]

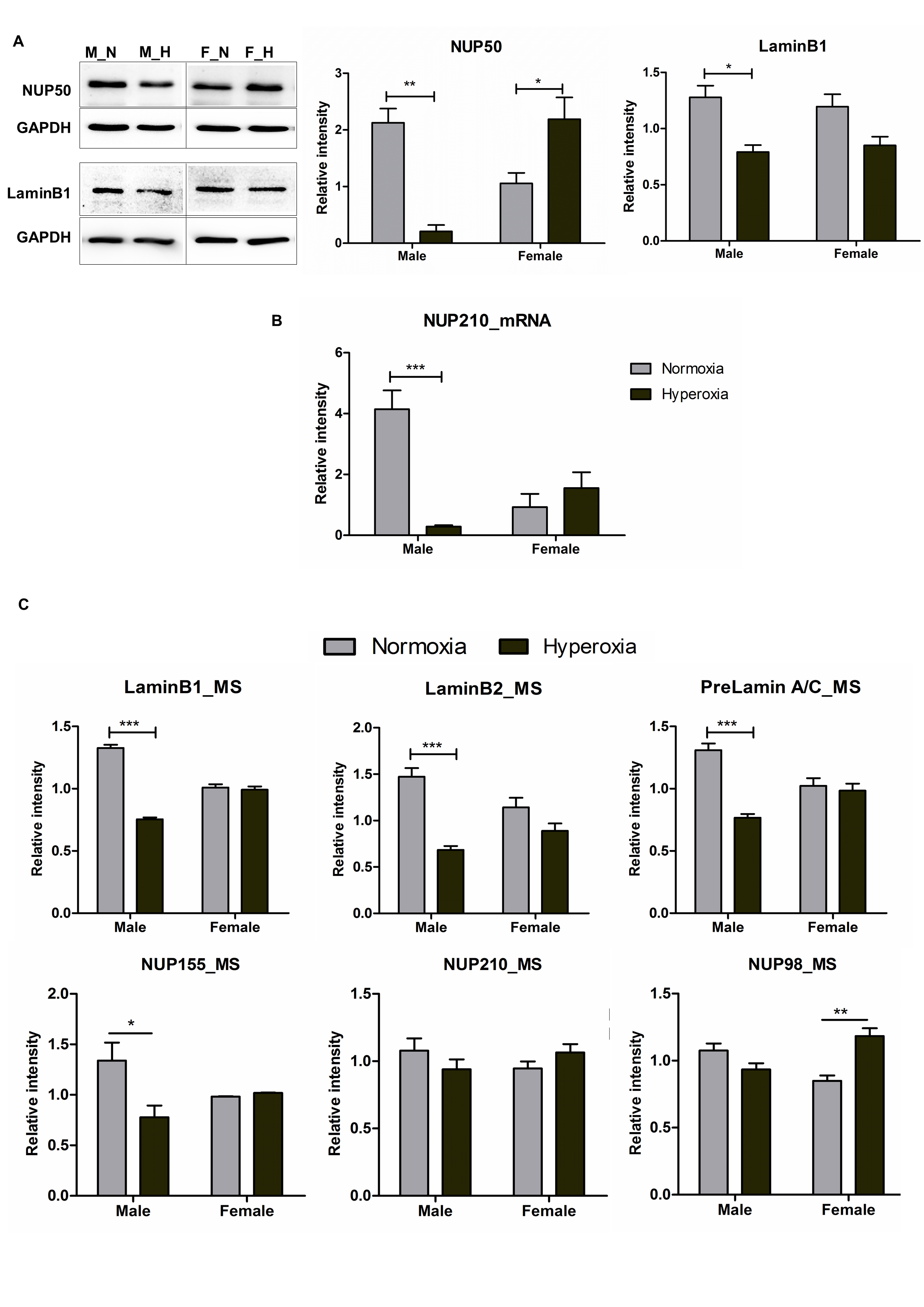

Supplement: Supplementary file 2 — Additional file 2: Figure S1. Proteins involved in cell adhesion and migration downregulated in male OPCs. (A) Immunoblot analysis of RhoA protein showing downregulation in male OPCs post 24 h 80%O2 treatment. (B) Heat-map representation of cell adhesion related proteins that were dysregulated in male and female derived OPCs post 24 h 80%O2 treatment in comparison to 3%O2 (normoxia) controls. Mapped expression ratios are depicted with a color scale as shown in the figure, such that highly downregulated proteins are indicated in red, intermediate in yellow, and highly upregulated proteins in green. Proteins are sorted according to Gene ontology (biological process). Dark outlined cells represent the significant proteins in each group. The cut off p value being 0.07. Data are representative of five independent experiments. (C) Independent intensities of Rac1, Mapk1, Map 2k1 and Arpc1b plotted from. MS results showing a significant downregulation in male derived-OPCs post hyperoxia. Data are representative of three experiments. Bars and error represent mean ± SEM of replicate measurements. ∗p < 0.05, ∗∗p < 0.01, ∗∗∗p < 0.001 (Student’s t test). Figure S2. Changes in nuclear envelope proteins in OPCs post hyperoxia. (A) Western blot analysis of male and female OPCs with anti-Nup-50 and anti-Lamin B1 antibodies under normal (3%O2) conditions and post 24 h 80%O2 treatment, showing a significant decrease in expression in the male OPCs. Whereas in female OPCs, Nup50 showed a significant upregulation post hyperoxia. ***p < 0.001, **p < 0.01, *p < 0.05 (Student’s t test), n = 3. Values are means ± SEM. (B) mRNA expression of Nup210 showing downregulation in male OPCs and upregulation in female OPCs post hyperoxia. ***p < 0.001, **p < 0.01, *p < 0.05 (Student’s t test), n = 3. Values are means ± SEM. (C) Intensities of Lamin B1, Lamin B2, Pre-Lamin A/C, Nup210, Nup155 and Nup98 plotted from the mass spectrometry results show a significant downregulation of Lamin B1, Lamin B2, [file 40348_2020_102_MOESM2_ESM.zip › Supplementary Figure S-2.tif]

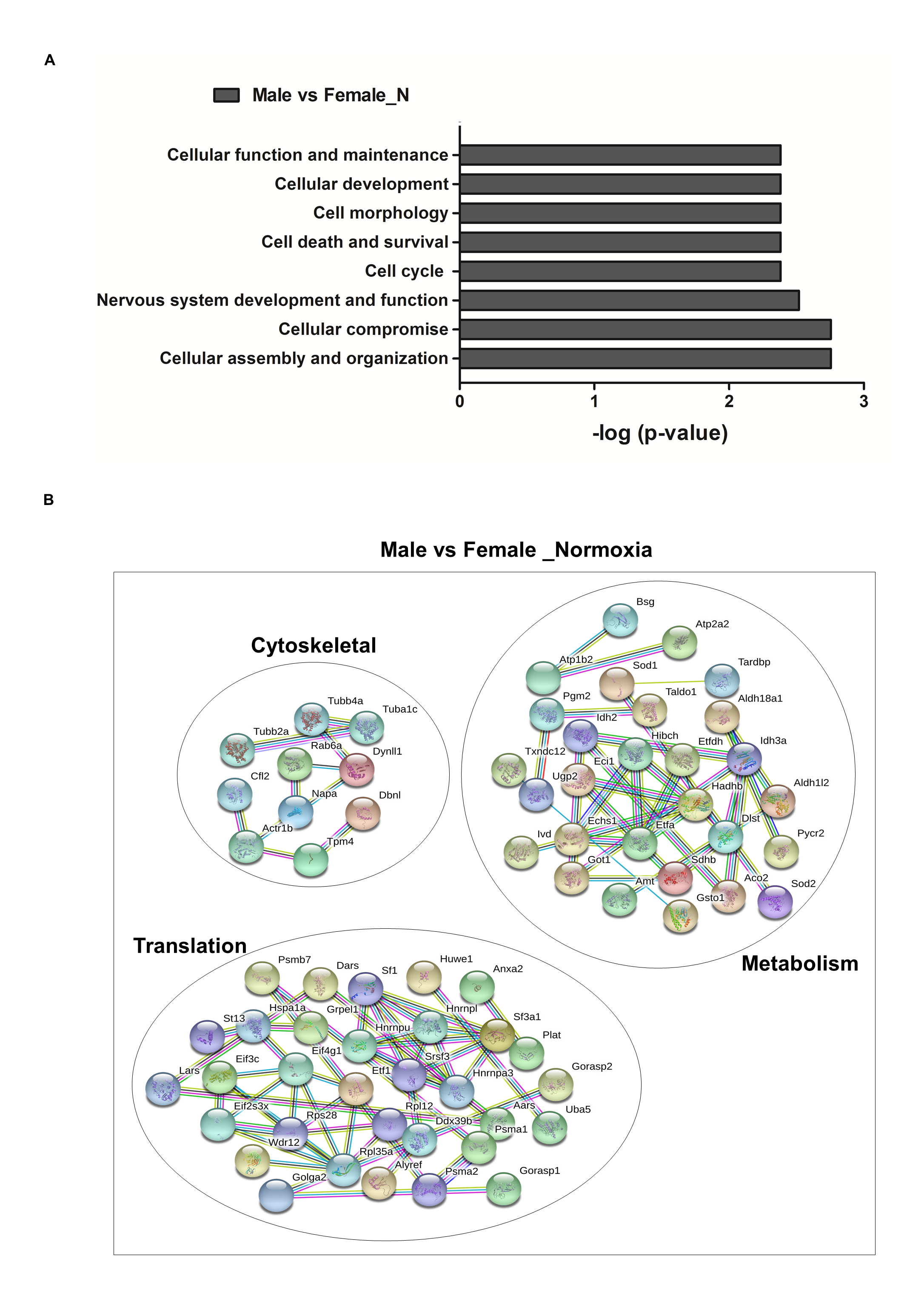

Supplement: Supplementary file 2 — Additional file 2: Figure S1. Proteins involved in cell adhesion and migration downregulated in male OPCs. (A) Immunoblot analysis of RhoA protein showing downregulation in male OPCs post 24 h 80%O2 treatment. (B) Heat-map representation of cell adhesion related proteins that were dysregulated in male and female derived OPCs post 24 h 80%O2 treatment in comparison to 3%O2 (normoxia) controls. Mapped expression ratios are depicted with a color scale as shown in the figure, such that highly downregulated proteins are indicated in red, intermediate in yellow, and highly upregulated proteins in green. Proteins are sorted according to Gene ontology (biological process). Dark outlined cells represent the significant proteins in each group. The cut off p value being 0.07. Data are representative of five independent experiments. (C) Independent intensities of Rac1, Mapk1, Map 2k1 and Arpc1b plotted from. MS results showing a significant downregulation in male derived-OPCs post hyperoxia. Data are representative of three experiments. Bars and error represent mean ± SEM of replicate measurements. ∗p < 0.05, ∗∗p < 0.01, ∗∗∗p < 0.001 (Student’s t test). Figure S2. Changes in nuclear envelope proteins in OPCs post hyperoxia. (A) Western blot analysis of male and female OPCs with anti-Nup-50 and anti-Lamin B1 antibodies under normal (3%O2) conditions and post 24 h 80%O2 treatment, showing a significant decrease in expression in the male OPCs. Whereas in female OPCs, Nup50 showed a significant upregulation post hyperoxia. ***p < 0.001, **p < 0.01, *p < 0.05 (Student’s t test), n = 3. Values are means ± SEM. (B) mRNA expression of Nup210 showing downregulation in male OPCs and upregulation in female OPCs post hyperoxia. ***p < 0.001, **p < 0.01, *p < 0.05 (Student’s t test), n = 3. Values are means ± SEM. (C) Intensities of Lamin B1, Lamin B2, Pre-Lamin A/C, Nup210, Nup155 and Nup98 plotted from the mass spectrometry results show a significant downregulation of Lamin B1, Lamin B2, [file 40348_2020_102_MOESM2_ESM.zip › Supplementary Figure S-3.tif]

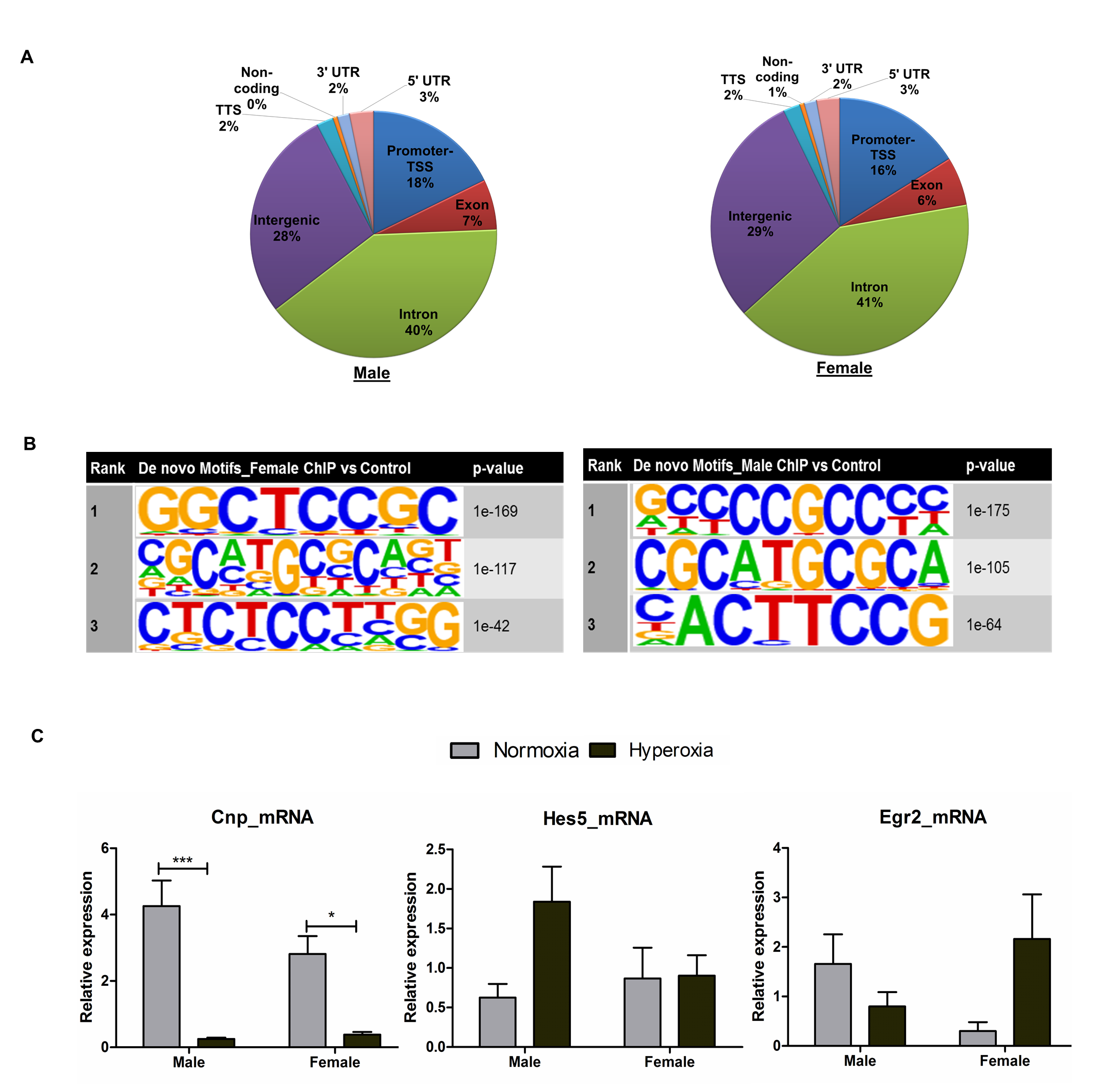

Supplement: Supplementary file 2 — Additional file 2: Figure S1. Proteins involved in cell adhesion and migration downregulated in male OPCs. (A) Immunoblot analysis of RhoA protein showing downregulation in male OPCs post 24 h 80%O2 treatment. (B) Heat-map representation of cell adhesion related proteins that were dysregulated in male and female derived OPCs post 24 h 80%O2 treatment in comparison to 3%O2 (normoxia) controls. Mapped expression ratios are depicted with a color scale as shown in the figure, such that highly downregulated proteins are indicated in red, intermediate in yellow, and highly upregulated proteins in green. Proteins are sorted according to Gene ontology (biological process). Dark outlined cells represent the significant proteins in each group. The cut off p value being 0.07. Data are representative of five independent experiments. (C) Independent intensities of Rac1, Mapk1, Map 2k1 and Arpc1b plotted from. MS results showing a significant downregulation in male derived-OPCs post hyperoxia. Data are representative of three experiments. Bars and error represent mean ± SEM of replicate measurements. ∗p < 0.05, ∗∗p < 0.01, ∗∗∗p < 0.001 (Student’s t test). Figure S2. Changes in nuclear envelope proteins in OPCs post hyperoxia. (A) Western blot analysis of male and female OPCs with anti-Nup-50 and anti-Lamin B1 antibodies under normal (3%O2) conditions and post 24 h 80%O2 treatment, showing a significant decrease in expression in the male OPCs. Whereas in female OPCs, Nup50 showed a significant upregulation post hyperoxia. ***p < 0.001, **p < 0.01, *p < 0.05 (Student’s t test), n = 3. Values are means ± SEM. (B) mRNA expression of Nup210 showing downregulation in male OPCs and upregulation in female OPCs post hyperoxia. ***p < 0.001, **p < 0.01, *p < 0.05 (Student’s t test), n = 3. Values are means ± SEM. (C) Intensities of Lamin B1, Lamin B2, Pre-Lamin A/C, Nup210, Nup155 and Nup98 plotted from the mass spectrometry results show a significant downregulation of Lamin B1, Lamin B2, [file 40348_2020_102_MOESM2_ESM.zip › Supplementary Figure S-4.tif]

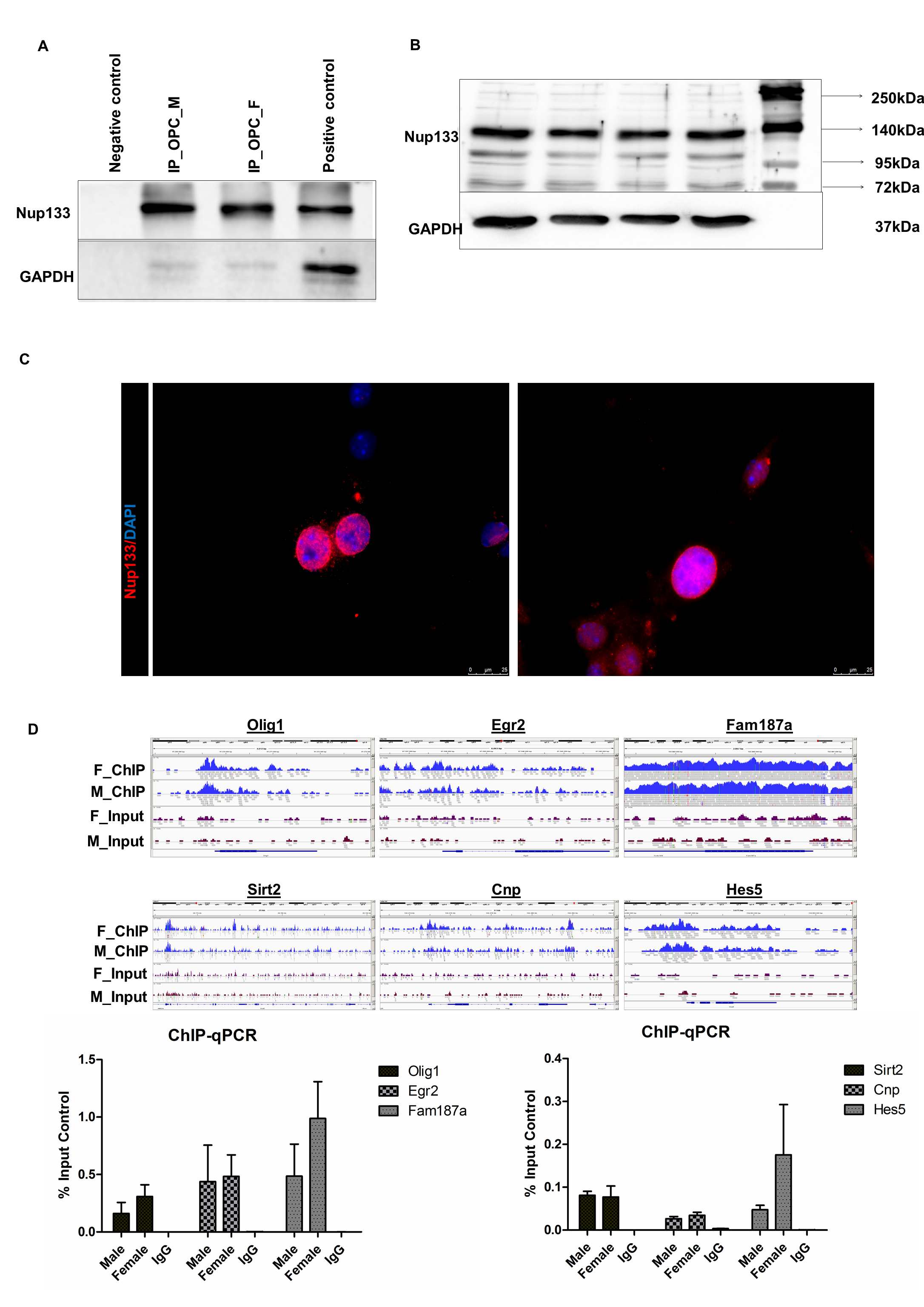

Supplement: Supplementary file 2 — Additional file 2: Figure S1. Proteins involved in cell adhesion and migration downregulated in male OPCs. (A) Immunoblot analysis of RhoA protein showing downregulation in male OPCs post 24 h 80%O2 treatment. (B) Heat-map representation of cell adhesion related proteins that were dysregulated in male and female derived OPCs post 24 h 80%O2 treatment in comparison to 3%O2 (normoxia) controls. Mapped expression ratios are depicted with a color scale as shown in the figure, such that highly downregulated proteins are indicated in red, intermediate in yellow, and highly upregulated proteins in green. Proteins are sorted according to Gene ontology (biological process). Dark outlined cells represent the significant proteins in each group. The cut off p value being 0.07. Data are representative of five independent experiments. (C) Independent intensities of Rac1, Mapk1, Map 2k1 and Arpc1b plotted from. MS results showing a significant downregulation in male derived-OPCs post hyperoxia. Data are representative of three experiments. Bars and error represent mean ± SEM of replicate measurements. ∗p < 0.05, ∗∗p < 0.01, ∗∗∗p < 0.001 (Student’s t test). Figure S2. Changes in nuclear envelope proteins in OPCs post hyperoxia. (A) Western blot analysis of male and female OPCs with anti-Nup-50 and anti-Lamin B1 antibodies under normal (3%O2) conditions and post 24 h 80%O2 treatment, showing a significant decrease in expression in the male OPCs. Whereas in female OPCs, Nup50 showed a significant upregulation post hyperoxia. ***p < 0.001, **p < 0.01, *p < 0.05 (Student’s t test), n = 3. Values are means ± SEM. (B) mRNA expression of Nup210 showing downregulation in male OPCs and upregulation in female OPCs post hyperoxia. ***p < 0.001, **p < 0.01, *p < 0.05 (Student’s t test), n = 3. Values are means ± SEM. (C) Intensities of Lamin B1, Lamin B2, Pre-Lamin A/C, Nup210, Nup155 and Nup98 plotted from the mass spectrometry results show a significant downregulation of Lamin B1, Lamin B2, [file 40348_2020_102_MOESM2_ESM.zip › Supplementary Figure S-5.tif]
